# Usability of End-to-End Encryption in E-Mail Communication

**DOI:** 10.3389/fdata.2021.568284

**Published:** 2021-07-14

**Authors:** Adrian Reuter, Ahmed Abdelmaksoud, Karima Boudaoud, Marco Winckler

**Affiliations:** ^1^ Department of Informatics, Technische Universität München, München, Germany; ^2^ Université Côte d’Azur, Polytech Nice Sophia, Nice, France; ^3^ I3S, CNRS, Université Côte d’Azur, Nice, France

**Keywords:** e-mail encryption, user study, user testing, usability, PGP, S/MIME, PEP

## Abstract

This paper presents the results of a usability study focused on three end-to-end encryption technologies for securing e-mail traffic, namely PGP, S/MIME, and Pretty Easy Privacy (pEp). The findings of this study show that, despite of existing technology, users seldom apply them for securing e-mail communication. Moreover, this study helps to explain why users hesitate to employ encryption technology in their e-mail communication. For this usability study, we have combined two methods: 1) an online survey, 2) and user testing with 12 participants who were enrolled in tasks requiring e-mail encryption. We found that more than 60% of our study participants (in both methods) are unaware of the existence of encryption technologies and thus never tried to use one. We observed that above all, users 1) are overwhelmed with the management of public keys and 2) struggle with the setup of encryption technology in their e-mail software. Nonetheless, 66% of the participants consider secure e-mail communication as important or very important. Particularly, we found an even stronger concern about identity theft among e-mail users, as 78% of the participants want to make sure that no other person is able to write e-mail on their behalf.

## Introduction

Nowadays, there are different security mechanisms allowing to protect the user privacy and to secure communication protocols. For example, Transport layer security (TLS) is one of the most prominent and widely adopted solutions for securing various protocols, particularly for browsing the World Wide Web. More recently, a huge step towards more secure Internet communication was achieved with the integration of end-to-end cryptography in mobile internet messenger services such as WhatsApp, Signal or Telegram. As far as electronic mail/e-mail is a concern, two major end-to-end encryption technologies have been available for decades, namely Pretty Good Privacy (PGP) (Atkins et al., 1996) and Secure Multipurpose Internet Mail Extensions (S/MIME) (Ramsdell, 1999). In addition to PGP and S/MIME, a recent initiative called Pretty Easy Privacy (pEp) (https://www.pep.security/) made efforts to simplify the usage of end-to-end cryptography in e-mail communication for novice users.

Despite the fact that, electronic mail is still one of the most commonly used communication channels, end-to-end encryption is only applied by a small fraction of e-mail users. According to Mathew Green (https://www.newyorker.com/tech/annals-of-technology/the-daunting-challenge-of-secure-e-mail), more than 95% of the overall e-mail traffic is exchanged without end-to-end encryption. Indeed, many users rely on unencrypted and unauthenticated e-mail communication without being aware of existing mechanisms that could mitigate and prevent the consequences of unsecure e-mail exchange. Applying end-to-end cryptography to our daily e-mail communication is considered crucial for four main reasons:

• Protect confidentiality;
• Protect privacy;
• Protect integrity;
• Provide authenticity and non-repudiation.

In this work, we try to identify usability issues that might hinder users from securing their daily e-mail communication using end-to-end encryption. Our study paid particular attention to the effectiveness dimension through the assessment of task complexity. To address this question, we have conducted a preliminary usability study that combines an online survey and direct observation of users during user testing of tools for e-mail encryption. The results presented in this paper are an extended version of the preliminary results published in the Financial Cryptography and Data Security Proceedings (Reuter et al., 2020). Hereafter we provide full details about the procedures and the data collected for the study and extended the analysis on the data. As we shall see, the tables and graphics were extended to better communicate the findings.

The rest of the paper is organized as follows. *E-Mail Encryption Technologies* briefly explains the three e-mail encryption technologies. *Methodology* presents the methodology used to conduct our usability study. *Results From the Online Survey, Results From the User Testing, and Discussion and Recommendations* discuss the obtained results. *Related Work* gives an overview about related work. Finally, *Conclusion* concludes this paper and provides an outlook on future work.

## E-Mail Encryption Technologies

This section provides an overview of the three encrypting technologies (namely, PGP, S/MIME, and pEp) that are subject of our study. Herein, we also present a brief analysis of the key management task from a user perspective.

### PGP

**PGP** is an encryption program that can be used for signing and encrypting e-mails, texts, files, directories, and whole disk partitions. It relies on public keys exchanged between users. There is no central trusted authority; users are responsible for finding, downloading and authenticating the public keys of other users. The authenticity of such entity-key-associations is asserted by the PGP users themselves who cross-sign each other's keys (Callas et al., 2007). Security and privacy are leveraged by a range of techniques (compression, public-key cryptography, symmetric cryptography, digital signatures, and the web of trust) that allow the users to send encrypted messages and check whether a message is authentic and has not been tampered with.

### S/MIME

**S/MIME** is an enhancement of the MIME standard (Borenstein and Freed, 1993), providing cryptographic security for e-mails based on MIME. Most popular e-mail clients such as MS Outlook, Mozilla Thunderbird or Apple Mail support S/MIME messages. S/MIME relies on the X.509 public key infrastructure (PKI) to exchange and validate the public keys of other e-mail users. Users must obtain a valid X.509 public key certificate signed by a trustworthy Certification Authority (CA). This digital certificate is then automatically embedded into all outgoing e-mails to distribute the public key of the sender. Thus, the e-mail software of users receiving an S/MIME-secured e-mail can extract the sender’s certificate including the embedded public key.

### pEp

**pEp** is based on Open PGP and its message format, but introduces several enhancements and new concepts to make e-mail encryption easier to use. The major improvement consists in automating procedures, as well as moving forward to a security by default instead of a security by opt-in philosophy. It is designed to work without prior configuration by the user (https://pep.foundation/docs/pEp-whitepaper.pdf). The own PGP key pair is automatically generated in background upon first usage of pEp, or imported automatically if the user previously used PGP and already has a key pair on her/his system. The own public key is always attached as a file to each outgoing e-mail, and consequently the public keys of other pEp users are received respectively by incoming e-mails (https://tools.ietf.org/html/draft-marques-pep-e-mail-02). Those public keys extracted from incoming e-mails are imported automatically into pEp. Using this key management approach, pEp does not depend on any centralized infrastructure, such as key servers or external certification authorities. To authenticate received public keys, pEp offers a so-called handshake: Once both users received an e-mail from the respective other user and thus having exchanged their public keys, pEp invites the users to compare the fingerprint of the received key. The fingerprints are mapped to natural language words and thus can be conveniently compared over an out-of-band process, e.g., a phone call or instant messaging (https://tools.ietf.org/html/draft-marques-pep-handshake-00).

### Key Management From a User Perspective

Each technology has its own strategy for implementing the key management process. The different strategies have a strong implication on the number of tasks (ex., pre-configuration tasks) that users have to go through before they are able to send or receive e-mails. In this section, we underline the differences, from a user point of view, between the three technologies with regard to key management. Specifically, we focus on the generation of key pairs (public and private keys) and retrieval and verification of the public keys of the recipients.

In PGP, users are responsible for generating their own key pairs and manage the public keys of other users (i.e., recipients), e.g., by searching through key servers or by asking the users directly.

With S/MIME, users must request a digital certificate signed by a trustworthy Certification Authority. By doing so, they implicitly obtain their key pair, contained in the certificate. The certificate/public key of the sender is always embedded into her/his outgoing e-mails (i.e., when sending an e-mail to a recipient). Likewise, when receiving an e-mail, the certificate/public key of the sender is imported in the Mail User Agent (MUA) automatically. As such, the certificate infrastructure masks the process of key management, which is achieved mostly without direct user interaction.

In pEp, the key management is done without user interaction. The key pair of the users is generated automatically when using pEp for the first time. The public key of the sender is always attached to outgoing emails, and thus the public keys of other users are extracted automatically from incoming emails and imported in the MUA. Moreover, compared to PGP and S/MIME, pEp offers an optional “handshake” procedure to authenticate received public keys (see *pEp*).

The number of tasks users have to perform allows measuring the effectiveness of interactive systems. As a rule of thumb, a system with fewer tasks is more effective as it requires less physical effort (fewer user interactions) and less cognitive effort (few information at each step to be treated by the user’s mind). Moreover, complex task sequences are more likely to introduce errors, which always reduce the user performance and cause dissatisfaction with the system.

## Methodology

To assess the usability of the three end-to-end encryption technologies, we have used two methods: an online survey with 50 participants and user testing with 12 participants.

For this preliminary study, we have targeted young (young adults) IT users (students and employees) to collect a first feedback about the knowledge, use, perception and concerns regarding PGP, S/MIME and pEp as they might be considered to have a higher level of IT knowledge than the average population. We have applied our survey using a convenience sample of participants issued from different countries, mainly Western Europe and North Africa. We analyzed whether there were differences in the responses depending on the respondent’s origin country.

Before designing the questionnaire for the online survey and defining the user testing protocol, we have conducted a preparatory analysis based on the inspection of the three e-mail encryption technologies at concern.

The details about the preliminary analysis, the online survey and the user testing are described hereafter.

### Analysis of Support of PGP, S/MIME, and pEp by Mail User Agents

The three technologies PGP, S/MIME, and pEp are dependent of the implementation and support given by the mail user agents (MUAs). As we shall see, not all currently available MUAs support these three technologies. Prior to the usability study, we have assessed the most commonly used MUAs to determine whether (or not) the MUA support the three e-mail encryption technologies (i.e., PGP, S/MIME, and pEp), how the technology is embedded into the MUA (native or *via* plugin) and what are the technical and usability challenges that other users might encounter when using these MUAs to secure their e-mails.

For this assessment, we conducted a systematic inspection of tools. The results of such inspections allowed us to estimate the time required by a knowledgeable user to perform tasks, as well as to anticipate the challenges that the participants might face when executing them. More specifically, we were able to:

• Identify which encryption technology is supported by which MUA;
• Prevent participants from testing MUAs that turn out to be unusable (e.g., due to discontinued development of encryption plugins, incompatibility of versions and operating system);
• Anticipate the challenges that participants might face when trying to use these three technologies in the context of a specific MUA.

Table 1 shows the MUAs considered in our analysis. It also depicts the plugin required to add PGP, S/MIME or pEp functionality to a MUA if not supported natively, if it has been tested in this preparatory step and pertinent remarks resulting from our analysis. In this table, we can see for example, that a plugin named Gpg4o has been implemented for PGP to be used with Microsoft Outlook, whereas the Enigmail plugin is another implementation of PGP that can be used for Thunderbird.

**TABLE 1:** Commonly used mail user agents (MUA) and their support for PGP, S/MIME and pEp.

Technology	Mail user agents	Plugin/Native	Native	Tested during the preparatory step	Our remarks
PGP	Outlook desktop 2013/2016	Gpg4o		Yes	• Plugin requires IMAP connection to work, which is not supported by the MUA (Outlook 2013)
					• Generate key pair
					• Interface distorted when writing e-mails
					• Automatically decrypt e-mails
	Windows 10 mail app	Not supported	Not supported	**-**	
	MacOS (Apple mail)	Not supported	Not supported	**-**	
	Thunderbird	Enigmail plugin		Yes	• Generate key pair and upload it to any server
					• Possibility to use an existing key pair
					• Automatically decrypt e-mails
	Gmail (webmail)	FlowCrypt		Yes	• Generate key pair and upload it to own server
					• Import keys from any server
					• Only sign or encrypt but not possible to both actions
	Other webmail	Mailvelope		Yes	• Searches for keys on one server at a time
					• Uploads own key on mailvelope server only
					• Not integrated directly into main window
	Apple iOS	iPG mail app		No	• Need to be paid. Thus not tested
	Android	Maildroid app and cryptoplugin		Yes	• Cryptoplugin requires managing keys
					• Not possible to create new keys, only import existing key
					• Maildroid app for sending and receiving secure mails
S/MIME	Outlook desktop 2013/2016		Native support	Yes	• Difficult to import e-mail certificate
					• Cannot initiate an encrypted conversation (2016). Possible to send an encrypted e-mail only as a reply to an encrypted e-mail
	Windows 10 mail app		Native support	No	• Only partially supported in combination with microsoft exchange only. This is why we have not tested it
	MacOS (Apple mail)		Native support	Yes	• Very easy to use, simply import certificate into MacOS.
	Thunderbird		Native support	Yes	• Difficult to import e-mail certificate
	Gmail (webmail)	Not supported	Not supported	**-**	
	Other webmail	No other webmail having a plugin for S/MIME	No other webmail supporting natively S/MIME	**-**	
	Apple iOS	Apple mail app		Yes	• Very difficult to configure
					• Different configuration for different iOS versions
	Android	Maildroid and cryptoplugin		Yes	• Cryptoplugin app is required for managing certificates
pEp	Outlook desktop 2013/2016	pEp for Outlook		No	• Need to be paid. Not tested
	Windows 10 mail app	Not supported	Not supported	**-**	
	MacOS (Apple mail)	Not supported	Not supported	**-**	
	Thunderbird	Enigmail		Yes	• Faulty key management in case of an existing PGP key
	Gmail (webmail)	Not supported	Not supported	**-**	
	Other webmail	No other webmail having a plugin for S/MIME	No other webmail supporting natively S/MIME	**-**	
	Apple iOS	App not yet released at time of study		No	• Not yet released. Thus, not yet tested
	Android	Official pEp app		Yes	• Considered easy to use

As we shall notice in Table 1, some e-mail encryption technologies are not available for some MUAs. Moreover, MUAs supporting the same encryption technology might presents some differences in terms of sequence of tasks to be completed by users. The analysis of MUAs allowed us to find some general issues with encryption mechanism:

• For PGP, we have identified several issues regarding mainly key management: The users must first generate the key pair, then upload the public key on a key server. With Mailvelope, it is not possible to search for a recipient’s public key on several servers at the same time, and the sender public key is only uploaded on Mailvelope’s proprietary key server. Moreover, for Android, it is not possible to generate new keys on-device, and with Microsoft Outlook, the interface is distorted when writing e-mails. However, it is possible to use PGP and import existing key pairs for Thunderbird and Google Webmail (Gmail).
• For S/MIME, we have noticed several problems, such as difficulty to import a certificate or initiate an encrypted e-mail exchange. For Apple Mail on MacOS, no particular difficulty has been identified by our users. For Apple Mail on iOS, our users perceived the configuration of S/MIME as difficult to do, as the configuration depends on rather hidden settings in the system menu, instead of settings in the mail app menu itself. Moreover, the configuration slightly varies for the different iOS versions.
• For pEp, only two MUAs could be tested and no major issue has been identified.

From the collection of MUAs listed in Table 1, we had to choose a subset of MUAs that could be used for **user testing**, taking into account the popularity of the MUAs and considering that each encryption technology should be tested on each major platform (if supported) and should be free of costs for our participants.

### Online Survey

An online survey was designed to collect information from a broad audience. The ultimate goal was to determine if participants were aware of email encryption technologies, whether (or not) they are using them, as well as their expectations and opinions on end-to-end encryption. The survey included closed-ended multiple-choice questions, open-ended questions, and rating questions using a Likert scale. The survey was divided in six parts, as follows:

**1. Presentation of the study**: This section presents the purpose of the study and includes questions for collecting demographic data, professional status of the participant, and security policies enforced by the employer of the participant.
**2. Relevance of e-mail exchange for the participant**: This section includes questions for identifying participant’s previous knowledge and experience in the field of e-mail security and her/his preferred mail user agent.
**3. Experience with PGP**: This section focuses on participant’s experience with PGP.
**4. Experience with S/MIME**: This section focuses on participant’s experience with S/MIME.
**5. Experience with pEp**: This section focuses on participant’s experience with pEp.
**6. Overall impression on e-mail security**: This section includes questions related to the perceived importance of e-mail security, and security issues previously experienced.

We would like to underline that most of the questions related to the three technologies were defined after testing (during the preparatory step) the different MUAs mentioned in Table 1. Moreover, for the questions related to the three different e-mail encryption technologies, we had similar questions to collect the feedback about the usage and preferences. However, there were some questions specific to each email encryption technology as there is a significant difference regarding key management (as elaborated on in *Key Management From a User Perspective*), in order to collect more specific feedback related for example on fingerprints (for PGP), certificates (for S/MIME) and handshakes (for pEp).

The survey was implemented using Google Forms and was open for responses for a duration of 2 weeks. The invitation to the survey was advertised mainly on Facebook in university student groups in Germany, Egypt and Morocco, but also using personal contacts. The participation in the study is anonymous. The results of this study are presented latter on in *Results From the Online Survey*.

### User Testing

A user testing has been done to allow the observation of users at work. The goal of the user testing was to focus on the effectiveness dimension through the assessment of task complexity at two levels: at the technology level (PGP, S/MIME and pEp) and at the MUA software level (as for each technology exist different implementations used in different MUA software). The user testing was important in order to get a more reliable and precise feedback from the participants regarding the challenges they could face when using these e-mail encryption technologies. Having this feedback allowed us to identify exactly which tasks represent a challenge for the participants and which aspects hinder them to use e-mail encryption in their daily e-mail exchange.

Regarding the MUA software tested, we have selected only the MUAs that we have already analyzed in the preparatory phase (see Table 1). Moreover, we assumed that even if the technology is generally usable, its implementation within an MUA might make it difficult to use and vice-versa. Therefore, we have decided to ask the participants to test either two different implementations (i.e. MUA software) of a same technology (either PGP, S/MIME or pEp) or two different e-mail encryption technologies. In the second case, we asked the participants to test a pEp implementation and then to choose between a PGP and S/MIME implementation, in order to have a feedback regarding pEp and check if it meets its goal of simplifying the e-mail encryption process.

The chosen MUAs are depicted in Table 2. In this table, we can see that each user has either tested two different MUA software for a same technology or two different technologies (with the same MUA or two different MUAs).

**TABLE 2:** Test scenarios and mail user agents tested by the users.

Tested technologies	E-Mail clients used	Test number
S/MIME + pEp	• Thunderbird (S/MIME)	Test #1
	• Thunderbird (pEp)	
	• Maildroid (S/MIME)	Test #2
	• Android (pEp)	
	• Outlook 2016 (S/MIME)	Test #3
	• Thunderbird (pEp)	
	• Outlook 2016 (S/MIME)	Test #4
	• Android (pEp)	
	• Outlook 2016 (S/MIME)	Test #5
	• Thunderbird (pEp)	
PGP + pEp	• FlowCrypt (PGP)	Test #6
	• Thunderbird (pEp)	
	• Maildroid (PGP)	Test #7
	• Android (pEp)	
	• Outlook 2016 (PGP)	Test #8
	• Thunderbird (pEp)	
S/MIME	• Thunderbird	Test #9
	• Outlook 2016	
	• Apple mail (iOS 12)	Test #10
	• Apple mail (MacOS desktop)	
PGP	• Thunderbird	Test #11
	• FlowCrypt	
pEp	• Thunderbird	Test #12
	• Android	

The user testing covered all the tasks necessary to encrypt emails, including installation (when required) and configuration of the encryption tools, and sending of a secure email. Participants have given written consent to participate in the study. The user testing protocol started with a short interview of the participant to collect participant’s demographic data (ex., age, nationality, profession, etc.), preferred mail user agent (MUA) or e-mail program used to read emails, and previous experience with cryptography (in general or e-mail encryption in particular).

After the interview, we proceeded with the observation of the user using a MUA to send secure e-mails. The aim of the user testing was to focus on the observation of the complexity of the tasks related to a specific encryption technology when using familiar MUA software, rather than to challenge the users with an unknown MUA. Therefore, we proposed to the participants to use their preferred MUA and focus on the configuration and use of encryption features to send a secure e-mail.

The participants were asked to enable and configure the security features to use a specific e-mail encryption technology and send a secure e-mail to the evaluator conducting the user testing. Very few information about how to accomplish this task was provided to the participants during the testing. For every task, we have assigned an expected completion time. This time was an estimation provided by the evaluators based on their usage of the MUA software. When a participant was struggling to perform a task beyond the expected time, the task was marked as a failure and the user received hints to complete the task. This help was important to allow users to continue with the next task. The user testing was completed once the evaluator received an e-mail sent by the participant that was successfully encrypted and signed. The participants used their own laptop for the test. Some participants performed the test remotely. For these participants, we have used a screen sharing solution to observe them and collect their feedback regarding the different tasks. The participants themselves were also asked to verbalize their personal impression about the usability of the technology under test, once the testing was completed.

Table 3 shows in detail the information given to the participants before starting the test.

**TABLE 3:** Testing protocol.

Step 1	Interview of the participants covering: Demographic information (age, nationality, profession), preferred mail user agent, and previous knowledge about cryptography and e-mail encryption	
Step 2 (if needed)	Installation of mail user agent and configuration of mail access through IMAP **with supervisor help**	
Step 3	Participants are asked to send an encrypted and signed e-mail to the supervisor using one of the following encryption technologies. The instructions were adapted according idiosyncrasies of the tools employed by the user as shown below	
	**PGP**	
	**Instructions**	For **FlowCrypt**: no further information
		For **Thunderbird**: install Enigmail plugin, activate setting “enforce PGP and S/MIME”
		For **Outlook**: install gp4o plugin
		For **Android**: install Maildroid (mail reader) and CryptoPlugin (key administration)
	**Goal**	To send encrypted and signed e-mail + compare key fingerprints
	**S/MIME**	
	**Instructions**	For **all**: Obtain a certificate from: https://extrassl.actalis.it/portal/uapub/free-mail?lang=en
		For **Thunderbird**: no further information
		For **Outlook**: go to “trust center settings” for importing the certificate
		For **Android**: install Maildroid (mail reader) and CryptoPlugin (certificate administration)
		For **Apple-mail**: no specific recommendation
	**Goal**	To send encrypted and signed e-mail
	**pEp**	
	**Instructions**	For **Thunderbird**: active “enforce pretty easy privacy” setting
		For **Android**: install pEp official Android app
	**Goal**	To send secure e-mail + execute handshake

The bold values to distinguish the names of the different mail user agent (MUA).

The results of the user testing are presented at *Results From the User Testing*.

## Results From the Online Survey

The online survey was launched on November 30, 2018 and closed with 50 participants on December 12, 2018 when we started the analysis of the results. As stated previously (*Methodology*), for this preliminary study, we have targeted young adults, from Egypt, Morocco and Germany, having an IT profile (students and employees working for IT organizations). We had also some participants from other EU countries. Hereafter, we provide the most relevant results regarding the six parts of the survey (see *Online Survey*).

### Feedbacks on Demographic Data

The majority of the participants were IT students (66%) under 30 years of age (88% between 21 and 28). Regarding the distribution per country we had 40% participants from Egypt (65% students and 35% employees), 26% participants from Morocco (53.85% students and 46.15% employees), 20% participants from Germany (90% students) and 14% participants from other EU countries. Overall, 57.15% of participants were students and 42.85% employees of IT companies.

### Feedbacks on Knowledge and Experience Regarding Encryption of E-Mail Exchange

**FIGURE 1:**
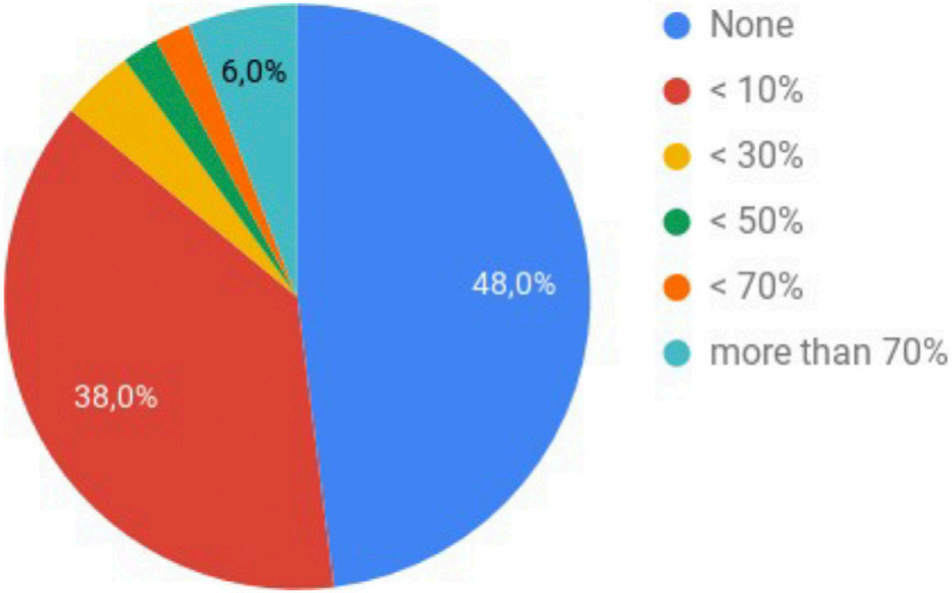
Answers to the question: *How many of the received e-mails are sent encrypted?.*

**FIGURE 2:**
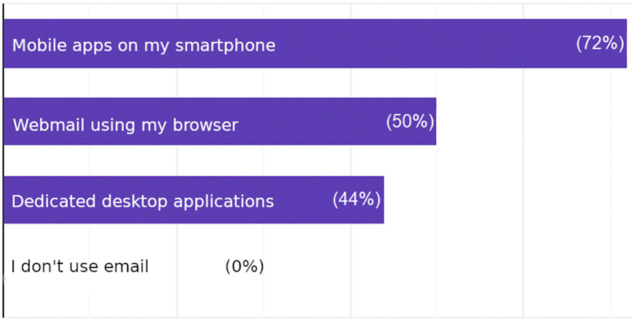
Answers to the question: Which mail software do you use to access your e-mails*?.*

The results concerning the personal experience of the participants with e-mail exchange showed that e-mail constitute a remarkable portion of their daily communication, reaching at least seven e-mails per day, but most of them were neither encrypted nor signed, 38% received or sent at least one email encrypted per day (see Figure 1), and less than half of the participants were obliged to use end-to-end encryption by their organization. Regarding the use of email software, the participants used more than one software. More than half of the participants used dedicated mobile applications, 50% used webmail, and 44% used dedicated desktop applications (see Figure 2).

Regarding the distribution per country there were no major differences for the number of e-mails exchanged per day. However, for the number of non encrypted/encrypted e-mails sent or received, there are some differences:

• For Egypt: 60% of e-mails are not encrypted, 30% of the participants receive less than 10% of e-mails that are encrypted, 10% of participants receive between 30 and 50% of encrypted e-mails.
• For Morocco: 38.46% of e-mails are not encrypted, 30.77% of the participants receive less than 10% of e-mails that are encrypted, 7.69% of the participants receive less than 30% of encrypted e-mails and 23.08% of the participants receive more than 70% of encrypted e-mails.
• For Germany: 50% of e-mails are not encrypted, 50% of the participants receive less than 10% of e-mails that are encrypted.
• For other EU countries: 28.57% of e-mails are not encrypted, 57.14% of the participants receive less than 10% of e-mails that are encrypted and 14.29% of the participants receive less than 70% of encrypted e-mails.

### Feedbacks on Knowledge and Experience With PGP, S/MIME, and pEp

#### Results for PGP

The results regarding the use of PGP are:

##### Knowledge Regarding PGP

60% of the participants never heard about PGP, 40% knew PGP but only 24% were also using it. From a country perspective:

• **Egypt**: 100% of the participants never heard about PGP.
• **Morocco**: 61.54% of the participants never heard about PGP and 38.46% knew PGP but never used it.
• **Germany**: 100% of the participants knew PGP and 80% use it (which means only 20% never used it).
• **Other EU countries**: 28.57% of the participants never heard about PGP, 71.43% knew PGP and 57.15% used it.

##### Experience With PGP

All the results provided below have been collected from European participants (i.e., from Germany and other EU countries), as Egyptian and Moroccan participants do not use PGP.

• 70% of the participants stated that they could not use PGP for all e-mails due to the fact that the recipient did not use PGP.
• 25% of the participants thought that it was difficult to find the recipient’s public key, 20% thought that configuring PGP was time consuming and just 5% declared that PGP is not implemented on their favorite platform/email software.
• 26.66% of the participants, who were using PGP, were always verifying the fingerprint of the recipient key, 40% were doing it occasionally, 20% never did and 13.33% did not know.
• The participants conceded that PGP guaranties privacy, confidentiality, authenticity and integrity, in addition to the fact that there was no cost for using it. However, they stated that comparing fingerprints was difficult and time consuming, and required the recipient to use it as well, which was not always the case given that PGP is not widely adopted.
• Participants suggested to make PGP supported on all platforms and simplify fingerprint comparison.

#### Results for S/MIME

The results regarding the use of S/MIME are:

##### Knowledge Regarding S/MIME

64% of the participants never heard about S/MIME, 36% knew it but only 18% were also using it. From a country perspective:

• **Egypt**: 95% of the participants never heard about S/MIME.
• **Morocco**: 69.23% of the participants never heard about S/MIME and 30.77% knew it but only 23.07% used it.
• **Germany**: 80% of the participants knew S/MIME and 40% used it.
• **Other EU countries**: we had similar results as for PGP. 28.57% of the participants never heard about S/MIME, 71.43% knew S/MIME and 57.14% used it.

##### Experience With S/MIME

The results presented below have been collected from all participants knowing and using S/MIME except Egyptian participants who do not use S/MIME.

• 11.11% of the participants were always sending e-mails using S/MIME, 55.56% were doing it occasionally, 22.22% were doing it upon request and 11.11% never did.
• 66.67% of the participants were receiving occasionally e-mails secured with S/MIME, 22.22% were receiving it upon request and 11.11% never received e-mails secured with S/MIME.
• 61% of the participants stated that the recipient was not using S/MIME.
• 28% did not trust digital certificates or its issuing entity and only 11% did not know how to obtain a digital certificate.
• 17% encountered difficulties configuring their environment to use S/MIME.
• 27% of the participants (in this result, we included participants from Egypt who knew S/MIME) admitted that they had issues with untrusted certificates. It was the case for only 12.5% of German participants, 25% of Moroccan participants but 40% of participants from other countries and 100% of Egyptian participants.
• 28% indicated that paying for a trustworthy certificate is an obstacle.
• The participants agreed that S/MIME had the advantage of being integrated in most email clients including Apple MacOS/iOS, but discredited it because they needed to pay to obtain a trustfully certificate.

#### Results for pEp

Regarding pEp, the results showed that it is not as known as PGP and S/MIME and only 10% knew it. No participant stated that she/he ever used it. From a country perspective, we had similar results:

• **Egypt**: 95% of the participants never heard about pEp.
• **Morocco**: 92.31% of the participants never heard about pEp.
• **Germany**: 90% of the participants never heard about pEp.
• **Other EU countries**: 85.71% of the participants never heard about pEp.

Moreover, 40% of the participants hesitated to use pEp because their recipients would not use it.

### Feedbacks on the Overall Impression on E-Mail Security

The goal of the last part of the survey was to gather the overall impression on end-to-end encryption, by scaling the degree of awareness of the participants on matters of e-mail exchange security, especially if they already encountered an e-mail privacy issue.

#### Importance of E-Mail Encryption

**FIGURE 3:**
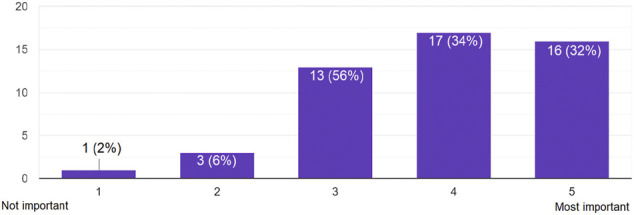
Answers to the question: *According to your personal impression, how would you scale the importance of e-mail encryption?*.

Assessing their overall impression, the participants were mostly aware of the importance of e-mail encryption: 66% thought that e-mail encryption is important to very important (34% for important and 32% for very important) (see Figure 3). From a country perspective, we had similar results:

• **Egypt**: 60% of the participants thought that e-mail encryption is important to very important (20% for important and 40% for very important).
• **Morocco**: 76.92% of the participants thought that e-mail encryption is important to very important (53.85% for important and 23.08% for very important).
• **Germany**: 60% of the participants thought that e-mail encryption is important to very important (40% for important and 20% for very important).
• **Other EU countries**: 71.42% of the participants thought that e-mail encryption is important to very important (28.57% for important and 42,85% for very important).

#### Feedback Regarding Potential Security Issues When Exchanging non Encrypted e-Mails

**FIGURE 4:**
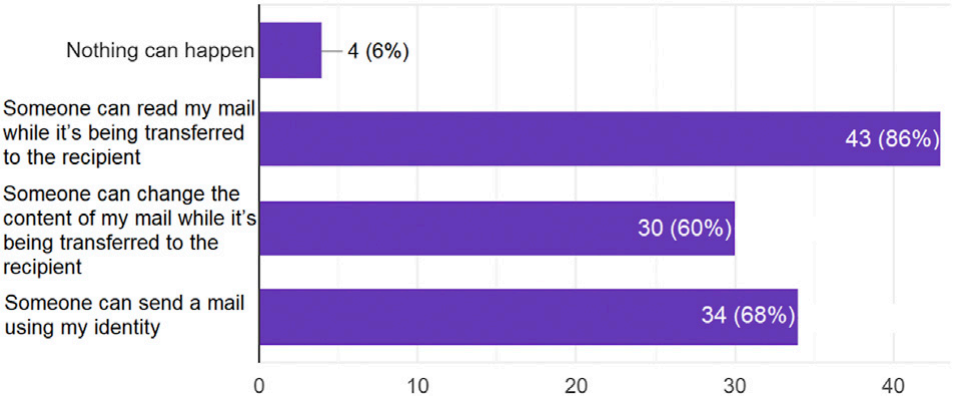
Answers to the question: *Considering a scenario of using non-encrypted e-mail communication, which of the following may occur?.*

Considering the scenario of non-secure e-mail exchange, more than 60% of the participants could imagine that their emails can be actively tampered with; and even a larger percentage of 86% assumed that an entity other than the e-mail recipient can read the email content (see Figure 4).

#### Feedback Regarding Security Properties

**FIGURE 5:**
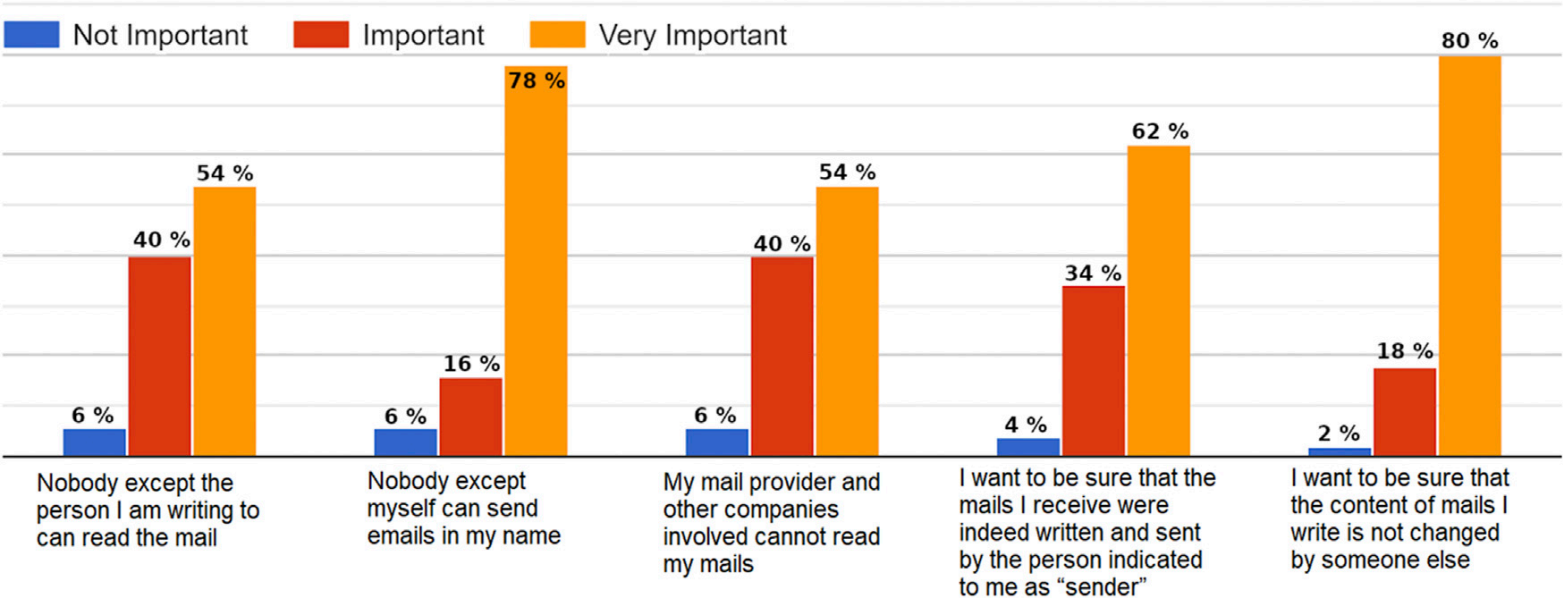
Answers to the question: *Please indicate the importance that the following security goals have for you.*

Assessing the importance of specific security goals, almost all of the participants estimated confidentiality, integrity and authenticity of their e-mails as important or very important. For only 6% of the participants, confidentiality did not matter and for only 2% the integrity of sent e-mails did not matter (see Figure 5). European and North African participants had similar concerns regarding the three security properties.

Comparing the feedback regarding the importance of security properties, we were surprised to notice that the users were more concerned about ***integrity*** (*80% thought that it is very important*) and ***authenticity*** (to be sure that nobody can spoof their identity and send emails in their name) than ***confidentiality***. This result is probably due to the fact that most of the participants were young adult students. It would be very interesting to conduct the same study with other category of people (teenagers and older users) to check if they have the same concerns. From a country perspective, we had similar results regarding each security property and the different questions.

**Integrity**: “I want to be sure that the content of e-mails I write is not changed by someone else”.

• **Egypt**: 75% of the participants thought that it is very important and 25% that is important.
• **Morocco**: 84.62% of the participants thought that it is very important. 7.69% indicated that is important and also 7.69% mentioned that is not important.
• **Germany**: 90% of the participants thought that it is very important and 10% that is important.
• **Other EU countries**: 71.43% of the participants thought that it is very important and 28.57% that is important.

**Confidentiality (regarding the recipient)**: “Nobody except the person I am writing to can read the e-mail”.

• **Egypt**: 45% of the participants thought that it is very important, 45% that is important and only 10% mentioned that is not important.
• **Morocco**: 61.54% of the participants thought that it is very important. 30.77% indicated that is important and only 7.69% mentioned that is not important.
• **Germany**: 70% of the participants thought that it is very important and 30% that is important.
• **Other EU countries**: 42.86% of the participants thought that it is very important and 51.14% that is important.

**Confidentiality (regarding third parties)**: My e-mail provider and other companies involved can not read my e-mails.

• **Egypt**: 45% of the participants thought that it is very important, 50% that is important and only 5% mentioned that is not important.
• **Morocco**: 61.54% of the participants thought that it is very important. 30.77% indicated that is important and only 7.69% mentioned that is not important.
• **Germany**: 40% of the participants thought that it is very important, 50% that is important and 10% that is not important.
• **Other EU countries**: 85.71% of the participants thought that it is very important and 14.29% that is important.

**Authenticity (no identity spoofing)**: Nobody except myself can send e-mails in my name.

• **Egypt**: 70% of the participants thought that it is very important, 20% that is important and only 10% mentioned that is not important.
• **Morocco**: 84.62% of the participants thought that it is very important. 7.69% indicated that is important and also 7.69% mentioned that is not important.
• **Germany**: 80% of the participants thought that it is very important and 20% that is important.
• **Other EU countries**: 85.71% of the participants thought that it is very important and 14.29% that is important.

**Authenticity (regarding the sender)**: “I want to be sure that the e-mails I receive were indeed written and sent by the person indicated to me as sender”.

• **Egypt**: 45% of the participants thought that it is very important, 50% that is important and only 5% mentioned that is not important.
• **Morocco**: 76.92% of the participants thought that it is very important.15.38% indicated that is important and 7.7% mentioned that is not important.
• **Germany**: 70% of the participants thought that it is very important and 30% that is important.
• **Other EU countries**: 71.43% of the participants thought that it is very important and 28.57% that is important.

### Synthesis of Survey Results

When analysing the survey results, we were astonished by two important facts regarding the knowledge of e-mail encryption technologies by IT users and the importance of some security properties. PGP and S/MIME were particularly not popular in North Africa (Egypt and Morocco) and for pEp, it was not known at all even in Europe. For the security properties, the users were even more concerned about authenticity and integrity than confidentiality independently from their origin country. This last result should be compared with the feedback of younger and older users.

## Results From the User Testing

Using a convenience sample, we recruited 12 participants for the user testing. The setup implied that users should perform a set of tasks using a mail user agent (MUA) and an encryption technology. Thus, every session featured a unique configuration of MUA and encryption technology (such as Thunderbird + PGP, Thunderbird + S/MIME, Outlook + PGP, etc). The combination of MUA and the encryption technologies at concern lead to a very large number of configurations. It was not possible to test all the configurations with every participant, but each participant tested two configurations in a user testing session. Given the fact that participants were allowed to choose the MUA they preferred to use during the test, some of the MUA were not tested at all. Table 4 shows the confounding matrix indicating the number of pairs (MUA/encryption technology) tested. As we shall see in Table 4, five users tried PGP, nine users tried S/MIME, and 10 users tried pEp. The number of how often a certain configuration was tested varies from a single user to up to six users at most.

**TABLE 4:** Confounding matrix of mail user agent (MUA) and encryption technology including the number of tests made.

Mail user agent	PGP	S/MIME	pEp
Apple mail (iOS 12)	-	1	-
Apple mail (MacOS desktop)	-	1	-
Flowcrypt (gmail webmail)	2	-	-
Maildroid (Android)	1	1	-
Outlook 2016 (desktop)	1	4	-
pEp app (Android)	-	-	4
Thunderbird (desktop)	1	2	6
**Total**	**5 tests with 4 MUAs**	**9 tests with 5 MUAs**	**10 tests with 2 MUAs**

Independently of the MUA and the encryption technology used, users were asked to complete three main tasks: configure the MUA, perform the key management and compose a secure e-mail. The key management task was required only for PGP, as this is done automatically for S/MIME (when requesting a certificate) and pEp (see *Key Management From a User Perspective*).

Whilst the overall user goal (i.e., sending a secure e-mail) is the same for all scenarios tested, the sub-tasks (i.e., the sequence of steps required to perform the task) vary according to the encryption technology and its implementation in an MUA software. Indeed, the number of sub-tasks might differ among the MUAs even when comparing the same encryption technology. This means that sub-tasks are dependent of a particular implementation of the encryption technology in a MUA software (example: in Thunderbird, Microsoft Outlook, etc.). We cannot at this point compare different MUAs software but we can compare effectiveness with respect to how many steps are required to complete (sub-) tasks. For example, in Table 7, regarding pEp, we can see that the task *Configuration* requires one single step (namely, “install pEp mail app”) with Android, while it requires two steps when using Thunderbird. In our analysis, we also report the issues found with each MUA.

Regarding the comparison of PGP, S/MIME and pEp, we can only address it with respect to the final goal of the user, which is to write a secure e-mail. At this level of abstraction, the three encryption technologies are comparable. We have used generic metrics of effectiveness, where task complexity is measured as number of steps (not including MUAs implementation of subtasks).

The details about tasks and subtasks for every MUA are given below in Table 5 (for PGP), Table 6 (for S/MIME) and Table 7 (for pEp). Notice that in all those tables, the cells painted in red indicate that users exceed the expected time to complete the tasks. These tasks are often associated with usability problems that will be explained in the following.

**TABLE 5:** Analysis of PGP tasks with four MUAs.

MUA	Outlook 2016 (desktop)	Thunderbird (desktop)	Flowcrypt (Gmail webmail)	Maildroid (Android)
Tasks	Subtasks			
Configuration	Install Gpg4o plugin	Install Enigmail plugin	Install flowcrypt browser plugin	Install cryptoplugin app from google PlayStore
		Activate PGP in settings menu		
		Enforce usage of PGP in account settings		
Key management	Generate key pair	Generate key pair	Choose passphrase	Generate key pair externally *via* PC software or online service
	Import key pair	Import key pair		Transfer externally generated key to mobile device
	Get recipient key	Get recipient key		Import key pair
				Get recipient key
Mail composition	Write a new secure e-mail	Write a new secure e-mail	Write a new secure e-mail	Write a new secure e-mail
	Verify if incoming e-mail is secure	Verify if incoming e-mail is secure	Verify if incoming e-mail is secure	Verify if incoming e-mail is secure

**TABLE 6:** User testing results for PGP regarding the number of users who tested an MUA and who failed for a specific sub-task.

MUA	# Users tested	Tasks	Subtasks	Failed
Outlook 2016 (desktop)	1	Configuration	Install Gpg4o plugin	
		Key management	Import or generate key pair	
			Get recipient key	1
		Mail composition	Write a new secure e-mail	1
			Verify if incoming e-mail is secure	
Thunderbird (desktop)	2	Configuration	Install Enigmail plugin	
			Activate PGP in settings menu	
			Enforce usage of PGP in account settings	1
		Key management	Import or generate key pair	
			Get recipient key	1
		Mail composition	Write a new secure e-mail	
			Verify if incoming e-mail is secure	
FlowCrypt (gmail webmail)	2	Configuration	Install FlowCrypt browser plugin	
		Key management	Choose passphrase	
		Mail composition	Write a new secure e-mail	
			Verify if incoming e-mail is secure	1
Maildroid (Android)	1	Configuration	Install cryptoplugin app from google PlayStore	
		Key management	Generate key pair externally *via* PC software or online service	1
			Transfer externally generated key to mobile device	1
			Import key pair	
			Get recipient key	
		Mail composition	Write a new secure e-mail	
			Verify if incoming e-mail is secure	

**TABLE 7:** Analysis of S/MIME tasks with five MUAs.

MUA	Outlook 2016 (desktop)	Thunderbird	Apple mail (MacOS desktop)	Apple mail (iOS 12)	Maildroid (Android)
Tasks	Subtasks				
Configuration	Get a certificate	Get a certificate	Get a certificate	Get a certificate	Get a certificate
	Unzip to get the pfx file	Unzip to get the pfx file	Unzip to get the pfx file	Unzip to get the pfx file	Unzip to get the pfx file
				Transfer pfx file to the mobile device	Install cryptoplugin
	Import a certificate	Import a certificate	Import a certificate	Activate S/MIME.	Transfer pfx file to the mobile device
				Import a certificate	Import a certificate
Mail composition	Write a secure e-mail	Write a secure e-mail	Write a secure mail	Write a secure e-mail	Write a secure e-mail
	Verify if incoming e-mail is secured	Verify if incoming e-mail is secured	Verify if incoming e-mail is secured	Verify if incoming e-mail is secured	Verify if incoming e-mail is secured

We emphasize that, with regard to the small number of user tests for some configurations, our testing does not intent to be statistically representative. Nonetheless it succeeds in indicating complex configuration tasks as well as accessibility issues (e.g., lack of technical understanding or terminology) in dealing with e-mail encryption technologies.

### User Testing Results for PGP

Table 5 summarizes the main findings of the user testing of PGP encryption technology using four MUAs: Microsoft Outlook 2016, Thunderbird, Flowcrypt (Gmail webmail) and Maildroid (Android).

As we can see in Table 5, there is a difference in terms of number of steps/sub-tasks and therefore task complexity, particularly for the Configuration and Key management tasks.

The Configuration task requires only one step (i.e., sub-task) for Microsoft Outlook, Flowcrypt and Maildroid, whereas for Thunderbird three sub-tasks are necessary to complete this task. In addition, the sub-task Enforce usage of PGP in account settings had some usability issue.

The Key management task requires only one sub-task with Flowcrypt, whereas in Microsoft Outlook and Thunderbird two sub-tasks (Generate a key pair/Import a key pair and Get recipient key) are necessary and even more sub-tasks 3) for Maildroid. In Maildroid, the generation of a key pair (related to the task Generate a key pair in Outlook and Thunderbird) requires two sub-tasks: Generate key pair externally *via* PC software or online service and Transfer externally generated key to mobile device, which is more tedious.

The Mail composition task has the same number of sub-tasks for all the MUA software.

Table 6 shows the number of users who had some usability issues and for which sub-tasks they had these issues. Hereafter, we explain the difficulties reported by the participants in the context of their respective MUA.

#### Outlook 2016

The participant who tested Outlook 2016 reported problems with following sub-tasks:

• *Install Gpg40 plugin*: It was difficult to find the correct plugin to download.
• *Get recipient key*: The user reported difficulties in finding and downloading the recipient public key on key servers. Further analysis from our side showed that this issue is due to Gpg4o’s port configuration. The default configuration of *Gpg4o* is to connect to a key server using an unusual port, which sometimes results in an empty response or a refused connection. It was not trivial for the user to identify this issue and accordingly go into the respective Outlook settings to change the port number to the one that is commonly used by a certain key server.
• *Write a new secure e-mail*: When composing a new secure e-mail and selecting the encryption and/or signature feature(s), the graphical user interface got distorted. Buttons, labels and text fields became misaligned and overlapped, making the user interface almost unusable and particularly difficult to assess whether the correct option was selected. Consequently, sending emails was pretty much impossible.

#### Thunderbird

After installing the *Enigmail* plugin, the user had to configure other options in the privacy settings of Thunderbird that were difficult to find. Specifically, this was the case for the tasks *Activate PGP* and *Enforce usage of PGP*.

Moreover, other sub-tasks turned out to be tiresome:

• *Activate PGP*: This task had to be applied for each e-mail account in the account settings.
• *Get recipient key*: This task was identified as difficult because Enigmail was looking for the missing recipient public keys on only one server at a time. It was up to the user to manually change the key server on which **Enigmail** searches for missing keys. This required patience and willingness to tediously change the key server settings until succeeding in retrieving the recipient public key from one of the servers on which the recipient published her/his key.

#### Flowcrypt

The participants did not face any issue with FlowCrypt and it turned out to be very easy to use. The only remark that has been reported is that Flowcrypt publishes the new public keys generated by its users on its proprietary key server only (i.e., Flowcrypt key server) and not on other well-known PGP key servers. Thus, for users of other MUAs it is potentially difficult to find the public keys of those recipients who generated their key pair using Flowcrypt. However, Flowcrypt itself automatically searches for missing recipient keys on other well-known PGP key servers.

#### Maildroid

The main problem encountered with Maildroid by the participants is related to the *Key management* task, more specifically regarding the generation of a key pair. Actually, with Maildroid, it is not possible to generate a PGP key pair directly on the mobile device. The key pair has to be generated externally (e.g., on a PC) and then be transferred back to the mobile device, by self-sending it *via* e-mail, or uploading it to a cloud and then downloading it on the mobile, or through a USB cable, which requires intense user interaction and downgrades the user experience.

#### Summary

Thanks to the user testing, we could observe that PGP requires many manual configuration steps until successful usage. We particularly saw this for the sub-task *Get recipient key* when the users tried to import the public keys of the recipients, which always turned out to be difficult or tedious for all our participants, regardless of the tested platform except with **Flowcrypt,** as this is done automatically (we can notice that this sub-task does not exist for **Flowcrypt**). This is due to the design principle of PGP to let full control to the users with respect to key management–which at the same time is demanding a basic understanding of asymmetric cryptography and PGP technology.

More specifically, regarding the different MUAs, PGP turned out to be less usable in **Outlook** and **Thunderbird**. **Outlook** had a heavily buggy UI implementation that made writing secure e-mail difficult. Finding recipient keys failed in several cases when trying to send new secure e-mails. With **Thunderbird**, retrieving recipient keys showed to be tedious, because the plugin was looking for the public keys on only one server at a time. **Maildroid** had also an important usability problem regarding the generation of PGP key pairs, as **Cryptoplugin** (the plugin used by **Maildroid)** cannot generate new PGP key pairs itself. A new key pair needs to be generated externally and then transferred and imported to the mobile device.

In contrast, **FlowCrypt** was the easiest tool to use, as the users could generate a new key pair with only few clicks and the users did not have to search for the recipient keys as this is done automatically and on almost all commonly used key servers. Thereby, **FlowCrypt** solved nearly all usability issues encountered by our participants with the other MUAs. However, **Flowcrypt** has two main drawbacks: Firstly, the key pairs that it generates are uploaded on its own key server only (i.e., Flowcrypt key server) that is unknown by other MUAs implementing PGP, which does not facilitate the import of public keys of FlowCrypt users by other users using a different MUA. Secondly, at the time of this study, **FlowCrypt was** only supporting Google webmail.

### User Testing Results for S/MIME

Table 7 summarizes the main findings of the user testing of S/MIME encryption technology using five MUAs: Microsoft Outlook 2016, Thunderbird, Apple Mail (MacOS), Apple Mail (iOS 12) and Maildroid (Android).

The first important information that we can notice in this table is that S/MIME does not require the *Key management* task, as the users do not have to generate a key pair nor have to struggle with finding recipient public keys. They just must obtain and install a certificate and thereby their key pair will be managed without requiring their intervention.

For the comparison between the MUA software in terms of sub-tasks, we can see, in Table 7 that **Outlook**, **Thunderbird** and **Apple mail (MacOS)** requires the same sub-tasks for the *Configuration* task. However, for **Apple mail (iOS12)** and **Maildroid** two more sub-task are required: *Transfer pfx file to the mobile device* and *Activate S/MIME* for **Apple Mail (iOS12)** and *Install Cryptoplugin* and *Transfer pfx file to the mobile device* for **Maildroid**. Moreover, the sub-task *Unzip to get pfx file* for **Apple Mail (MacOS)** and *Transfer pfx file to the mobile device* for both **Apple Mail (iOS12)** and **Maildroid** were more difficult for the users.

Regarding the *Mail composition* task, all the MUAs have the same number of sub-tasks to complete this task.

The participants faced several difficulties that are explained below, depending on the MUA used.

In Table 8 we can see the number of users who faced some problems and for which sub-tasks they had these problems.

**TABLE 8:** User testing results for S/MIME regarding the number of users who tested an MUA and who failed for a specific sub-task.

MUA	# Users tested	Tasks	Subtasks	Failed
Outlook 2016 (desktop)	5	Configuration	Get a certificate	
			Unzip to get the pfx file	
			Import a certificate	
		Mail composition	Write a secure e-mail	4
			Verify if incoming e-mail is secure	1
Thunderbird (desktop)	3	Configuration	Get a certificate	
			Unzip to get the pfx file	
			Import a certificate	
		Mail composition	Write a new secure e-mail	
			Verify if incoming e-mail is secure	
Apple mail (MacOS desktop)	1	Configuration	Get a certificate	
			Unzip to get the pfx file	1
			Import a certificate	
		Mail composition	Write a new secure e-mail	
			Verify if incoming e-mail is secure	
Apple mail (iOS 12)	1	Configuration	Get a certificate	
			Unzip to get the pfx file	
			Transfer pfx file to the mobile device	1
			Activate S/MIME.	
			Import a certificate	
		Mail composition	Write a new secure e-mail	
			Verify if incoming e-mail is secure	1
Maildroid (Android)	1	Configuration	Get a certificate	
			Unzip to get the pfx file	
			Install cryptoplugin	
			Transfer pfx file to the mobile device	1
			Import a certificate	
		Mail composition	Write a new secure e-mail	
			Verify if incoming e-mail is secure	

#### Outlook 2016

The participants have faced some issues concerning the following sub-tasks:

• *Import a certificate*: It was difficult for the users to import their own digital certificate and there were no instructions to find where to import the pfx file. The respective configuration button was deeply hidden in the application’s settings menu. The participants passed too much time looking for the button in the settings.
• *Write a secure e-mail*: The participants experienced a strange bug. The users could encrypt their outgoing e-mails only as reply to another encrypted e-mail already received, but they could not encrypt a new e-mail even though they already retrieved the certificate of the recipient.
• *Verify if incoming e-mail is secure*: The icon showing that an e-mail is secure is very small and at the top right.

#### Thunderbird

The usability problems found with Thunderbird did not prevent the users to perform the tasks in the expected time. The sub-task *Import a certificate* was perceived as difficult but could be completed within the expected time.

#### Apple Mail (MacOS Desktop)

The user who has tested Apple Mail for MacOS has experienced two problems regarding the following sub-tasks:

• *Import a certificate*: The settings do not explain where to import the certificate (pfx file).
• *Unzip to get the pfx file*: This task exceeded the expected time. The user was not able to extract the pfx file from zip file and did not know what to do with the pfx file. Actually, MacOS does not support ZIP archives by default, which showed to be a major obstacle.

#### Apple Mail (iOS 12)

The user who has tested Apple Mail for iOS 12 reported problems with the following sub-tasks:

• *Unzip to get the pfx file*: This task was challenging for the user, as he could not unzip on his smartphone. After having received an archive file from the issuing Certification Authority *via* e-mail, the user had to decompress the archive file, containing the requested certificate, on a computer.
• *Transfer pfx file to the mobile device:* The only way for the user to transfer the certificate (the.pfx file) was to send it to himself by e-mail (as an attachment).
• *Import a certificate*: The user could not import the certificate if he did not first activate S/MIME manually in the system settings of the iPhone, which is not obvious.
• *Activate S/MIME*: The user spent a lot of time looking for the respective button, since it was hidden in the phone settings menu rather than in the application settings menu.
• *Write a secure e-mail*: The user had to import the certificate of the recipient manually.

#### Maildroid (Android)

The user who tested Maildroid had the same difficulties than the ones who used Apple Mail for iOS12 regarding the sub-tasks *Unzip to get the pfx file* and *Transfer pfx file to the mobile device*. He found difficult to not be able to get the certificate extracted directly on his smartphone and to be obliged to decompress the received certificate file using his laptop.

The other usability concern reported by the user is related to the task *Write a secure e-mail*, where the user had to use two different apps: Maildroid for sending the e-mail, and Cryptologin to encrypt/decrypt an e-mail and manage certificates.

In addition, the user was prompted for a passphrase every time he launched the crytoplugin app.

## Summary

Overall, S/MIME was considered easy to use on desktop MUAs except for **Outlook**. S/MIME does not require to deal with key management manually, making it easier to use specially for novice users. The only thing users are required to know, is how to apply for a digital certificate and how to import the certificate into the MUA. Once a user receives a signed e-mail, the sender’s public key is integrated automatically into the MUA. Consequently, the users do not have to perform any supplementary tasks other than configuring S/MIME in their desired MUA.

Using S/MIME with **Outlook** turned out to be less usable than **Thunderbird** and **Apple Mail** for **MacOS,** particularly in regard of importing an certificate, since the option was difficult to find by the users and it was not possible to send an encrypted email (outgoing encrypted e-mail) to a recipient who did not already previously send an encrypted e-mail (i.e., incoming e-mail already encrypted using S/MIME).

Regarding mobile MUAs, i.e., **Apple Mail (iOS 12)** and **Maildroid**, the main usability issue that bothered the users concerned the management of their own certificate that could not be received and unzipped directly on the mobile phone. The users had to extract the certificate (pfx file) on their computer first and send it back to their smartphone by e-mail in order to import it. Moreover, for **Apple Mail (iOS 12)**, it was very difficult to perform the task *Activate S/MIME,* as the button related to this task counter-intuitively is hidden in the phone settings menu instead of the settings menu of the mail app. Moreover, the way to activate S/MIME on iPhones varies from one iOS version to another.

### User Testing Results for pEp

Table 9 summarizes the main findings of the user testing regarding pEp encryption technology using two MUAs: pEp app for Android and Thunderbird. In this table, we can already see (like for S/MIME) that pEp requires only two tasks: *Configuration* and *Mail composition*. The *Key management* task does not exist as this is managed automatically (i.e., by design) by pEp. In general, pEp requires less steps/sub-task than PGP and S/MIME to configure it and use it. Thus, it is more effective in terms of task complexity.

**TABLE 9:** Analysis of pEp tasks with two MUAs.

MUA	pEp app (Android)	Thunderbird (desktop)
Tasks	Subtasks	
Configuration	Install pEp mail app	Install Enigmail plugin from add-on browser
		Force using pEp
	Perform handshake by communicating and comparing the trust words out of band	Perform handshake by communicating and comparing the trust words out of band
Mail composition	Write a new secure e-mail	Write a new secure e-mail
	Verify if incoming e-mail is secured	Verify if incoming e-mail is secured

The comparison of trust words through the so-called pEp handshake, in order to establish trust in the recipient key, was considered as convenient and rather easy step to do by most of our participants. Nevertheless, as we can see in Table 10, some users faced some usability issues. More specifically, one participant did not understand why such handshake is necessary and what to do with the trust words shown on the graphical user interface during the handshake procedure. Moreover, two users struggled with activating pEp in the configuration menu of Thunderbird.

**TABLE 10:** User testing results for pEp regarding the number of users who tested an MUA and who failed for a specific sub-task.

MUA	# Users tested	Tasks	Subtasks	Failed
pEp (Android)	3	Configuration	Install pEp mail app	
			Perform handshake by communicating and comparing the trust words out of band	1
		Mail composition	Write a secure e-mail	
			Verify if incoming e-mail is secure	
Thunderbird (desktop)	4	Configuration	Install Enigmail plugin from add-on browser	
			Force using pEp	2
			Perform handshake by communicating and comparing the trust words out of band	
		Mail composition	Write a new secure e-mail	
			Verify if incoming e-mail is secure	

### Synthesis of User Testing Results

When comparing the three e-mail encryption technologies considering the effectiveness dimension, we can firstly conclude that S/MIME and pEp are less complex as the users do not have to deal with their key pair, as the *Key management* task is required only for PGP. With PGP, most of the usability issues concern the sub-task *Get recipient key* (except for Flowcrypt where this is done automatically), particularly for desktop MUAs (Outlook and Thunderbird). Flowcrypt turned out to be the most usable MUA for PGP. With S/MIME, even if it was perceived as conceptually easier to use than PGP, the users faced some specific usability issues, particularly with Outlook and iOS Mail. Importing a user’s personal certificate required tedious search for the respective configuration item in the setting menus. In addition, due to an unresolvable software bug in Outlook, a user could send encrypted e-mail only as reply to encrypted incoming e-mails and not initiate a new secure e-mail communication.

Regarding mobile MUAs, the users encountered similar usability issues with PGP (the generation of a new PGP key pair has to be done externally, then the keys must be transferred to the mobile phone) and S/MIME (management of the certificate that could not be received and unzipped directly on the mobile phone).

pEp showed to be the easiest technology to use, but unfortunately it is not (yet) compatible with all major platforms. As a consequence, we could not test it on Apple MacOS or Apple iOS platforms, which are used by a large fraction of e-mail users.

## Discussion and Recommendations

The online survey allowed us to collect self-reported information about the use of the three e-mail encryption technologies (PGP, S/MIME and pEp). Such kind of declarative information cannot be verified in an online survey and thus we have to trust the participants’ comments during the analysis. Results were given by IT students and employees, who might be considered to have a higher level of knowledge about technology than the average population. Nonetheless, we have learned that PGP and S/MIME are not really known by North African users, which is surprising, as PGP exists decades. Moreover, another surprising result was the fact that people (particularly young adults) are more concerned by integrity and authenticity (particularly the fact that nobody can send an e-mail in their name) of their e-mails, rather than confidentiality.

User testing allowed a direct observation of the users, thus making this type of results more reliable then self-reported information, since they are based on observed phenomena. In fact, we were able to: 1) correlate some answers of the online survey with the results of the user testing and 2) to validate if the participants of the online survey criticize the same usability issues as the participants of the user testing. Moreover, thanks to the user testing, we gathered more specific usability issues regarding the different implementations of the three e-mail encryption technologies in the context of MUA software. Whilst user testing allows to find valid usability problems (as encountered by real users), these finds cannot be analyzed statistically due to the small number of participants. We acknowledge that this clearly is a limitation, because we cannot perform a deeper analysis on few observations.

The participants who answered encryption technology specific questions in the online survey have to be considered experienced using that respective technology and at least having some knowledge on security mechanisms. In contrast, most of our participants in the user testing never heard about PGP, S/MIME or pEp technologies before the test. However, we noticed that both participant groups encounter the same issues, and as such strongly confirm both independent results of our preliminary user study. For instance, the major obstacle for the participants of the online survey who stated to use PGP is retrieving the recipient key (25%). This showed to be the same major issue for the participants of the user testing. Moreover, the main issue for the participants of the online survey knowing and using S/MIME is to configure their environment to use S/MIME (17%), which has been confirmed in the user testing. Actually, when we helped the participants of the user testing by pointing them towards a free certification service, the follow-up issue was configuring their environment to use S/MIME (i.e. import own certificate). Finally, we noticed that all three technologies show low popularity. The online survey revealed that 60% never heard of PGP, 64% never heard of S/MIME and 90% never heard of pEp. In the user testing only one out of the twelve participants knew and used PGP (only one of three technologies). However, we believe that if the central usability issues carved out during this preliminary usability study are addressed by future releases of their implementations, user acceptance as well as the popularity of these technologies will increase. Based on these results we can suggest some improvements for each technology:

**Improvements for PGP:** We found that on several platforms, users are restricted to search for the recipient key only on one key server at a time, which makes finding and importing the recipient key one major obstacle when using PGP. Thus, we suggest fixing this issue by letting implementations search for the recipient key on multiple commonly known key-servers, without the user needing to manually adjust the key server setting for key import. Likewise, PGP implementations that generate new keys for their users should offer to publish the new key on all available key servers as default option, so that keys are more likely to be found by other e-mail users. We also suggest the usage of less tech-savvy language in user interfaces.

**Improvements for S/MIME:** We suggest Certification Authorities to provide further information on how to import the certificate into the most frequently used e-mail programs alongside the requested digital certificate. Also, we suggest integrating S/MIME support into webmail platforms, as webmail nowadays is used more commonly than dedicated desktop applications.

**Improvements for pEp:** We suggest pEp implementations to add further explanation about why doing the pEp handshake is important and what to do with the trust words displayed to the users during the handshake. We furthermore advise to briefly explain the color scheme that represents the security status of a communication channel to the user. Moreover, we criticize the design choice of not being able to re-do a new handshake with a user whose public key was previously mistrusted. A trust word mismatch can occur easily, e.g., due to a different language setting of the trust word dictionary on side of both users during handshake, and thus might result in an irreversible failed pEp handshake.

## Related Work

Even if PGP is a very secure mechanism and not easy to break, it has some well known usability issues. In particular, Bicchierai (2015) revealed that even PGP inventor Zimmerman encountered some difficulties in 2015 for decrypting an email he had received because he did not have the right PGP version on his device. The first usability evaluation for PGP and email security has been conducted by Whitten and Tygar (1999) 1999. This study focussed on usability of PGP 5.0 and has revealed serious usability problems of PGP for cryptography novice users. Another usability study has been done for PGP 9.0 by Sheng et al. (2006), where the automatic encryption has resolved some usability issues, but discovered that the users were not sure if their emails have actually been encrypted because the encryption was so transparent.

Garfunkel and Miller (2005) have created a secure email system using S/MIME called CoPilot, where key generation, key management, and message signing are done automatically. For the user study, the authors have followed a modified version of the study proposed by Whitten and Tygar (1999) and the email system has been used with Outlook express. Even if their study has confirmed an improvement regarding usability of S/MIME, it has also emphasized that users still encounter some difficulties “*with encrypting sensitive messages for the correct recipients*”.

Roth et al. (2005) strived to tackle the usability issue by carefully designing user interactions with encrypted email, more specifically proposing a non-intrusive design approach for secure emails considering the best trade-offs between usability and security for non-commercial users of email.

Kapadia (2007), who found that PGP was not usable with non-technical users, focussed on analysing usability of S/MIME and underlined some barriers that prevent users from using S/MIME to secure their emails. He has identified these barriers based on his own case study (and feedback) and not a specific user study.

Gerck (2008) conducted an analytic usability and security comparison of four technologies (PGP, X509/PKI, IBE and ZMAIL), in an enterprise use environment, focussing on four secure email tools available on the market (Outlook, Voltage, Hushmail, Message Guard) and considering a list of desirable features and shortcomings. This analysis did not focus on specific user groups nor a user study. Neppe (2008) completed Gerck’s results by providing his own feedback regarding Zmail.

Farrell (2009) analysed the security features of mail user agents (MUA) (no Webmails/Web-based mail clients), specifically focussing on end-to-end security and underlining that even if PGP (OpenGPG) and S/MIME are deployed they are not really used. He then tried to understand the users’ needs regarding email security starting from the statement that the designers of MUAs didn’t really ask the users what they really need and want. He concluded that the industry is continuing to make the same mistakes for new MUAs regarding the security mechanisms implemented whereas, it should “*go back to the basics and ask what the users really want - the ability to occasionally encrypt an email without much trouble at all*”.

Fry et al. (2012) identified S/MIME usability issues for three email clients, not considered in previous studies (Apple Mail, Thunderbird and Evolution), using a cognitive walkthrough methodology and defining fictional personas and scenarios.

Hof (2013) defined some guidelines to enhance usability of security mechanisms. Using these guidelines, he took as an example email security and more specifically email encryption to identify usability issues in GPGMail. Then, he applied the guidelines to design a more usable email encryption solution focussing on automated key management and trust management. The author evaluated the compliance of the proposed email encryption system with the usability guidelines. No user study has been conducted.

Moecke and Volkamer (2013) analyzed different e-mail services, defining security, usability and interoperability criteria and applying them to existing approaches. According to their results, closed and web-based systems like Hushmail are more usable, contrarily to PGP/SMIME, which require add-on or plug-in solutions for secure key management.

Ruoti et al. (2013) proposed a secure webmail system, called Pwm, that allows automatic encryption and key management. The first user studies conducted on this system revealed that having an automatic encryption can lead the users to make mistakes and generate some doubts about the real security of the system. The authors had then to conduct another user study with a manual encryption version of the email system, which has shown that the users have more trust in the system and make less mistakes.

In this work, the authors focussed on designing a new encryption email tool rather than analysing existing MUAs. The proposed tool has been used with gmail and Google Chrome Web browser and the user study targeted college students and gmail users only.

Following the user study, conducted in 2013, Ruoti et al. (2016) decided to conduct a new usability study following a new approach by involving pairs of novice users instead of the usual single user studies. In this study, the authors focussed on an improved version of Pwn, the webmail tool they have designed (that can be integrated with gmail) but also two other email tools Tutanota (depot-based tool) and Virtru (hybrid tool that has a depot-based version but also a plugin that can be integrated with gmail). The study has confirmed previous results, i.e. users trust less systems that hide security mechanisms and prefer integrated solutions and in this case compared to depot-based solutions. This study has also identified that users are interested by securing their emails but they do not really know when they could use it. In addition, only some of them think to use it on a regular basis. This study has the same limitations as previous studies regarding the user groups as it has focused on students and gmail users.

In (Ruoti et al., 2018), Ruoti et al. conducted the first comparative usability study of three key management schemes in secure e-mail tools. The results of the study showed that each key management strategy has its potential to be successfully used in secure e-mail. In (Ruoti et al., 2019), Ruoti et al. conducted another usability study regarding four e-mail tools that provide security for webmail. Their results indicated that the usability of e-mail encryption software largely depends on whether or not a well-designed tutorial is available. The study concluded that PGP seems unusable for novice users, mainly due to the user interaction with public key cryptography necessary to employ PGP.

In his thesis, Andersen (2016) analysed the usability of three key management mechanisms (PGP, IBE and passwords) by implementing them using MessageGuard and taking into account the results from previous studies to design an usable secure email. The user study conducted on the designed secure email system confirmed the results of previous works and suggested that PGP can be used by novices. The system has been used with gmail only and tested by gmail users and college students.

In (Ruoti and Seamons 2019), Ruoti and Seamons focussed on proposing future research work directions. They advocate for longitudinal studies that examine usability of existing secure email solutions in long-term, and which could reveal other usability issues that are unknown up to now. They also advise for putting effort into finding a usable key management procedure and in sensitizing users for the importance of email security and educating them in using email cryptography. Finally, they highlight the importance of meaningful UI design (e.g., they advocate for tight integration of secure mailing functionality into existing, non-secured mail functionality). In their paper, the authors underline “*For ordinary users, research has found mixed feelings regarding the necessity of secure email. [...] users do not sufficiently understand how encryption works or how it protects them, limiting the urgency they feel in adopting secure email.*” These findings support our approach, where we have tried to confront users with the potential consequences of compromised mail (such as other users can write emails in their name—Answer to the question “someone can send a mail using my identity,” ...), and lead to a drastic and clear response that the users want to be protected against such potential attacks.

Atwater et al. (2015) conducted a new user study contradicting the results of the previous study of Ruoti et al. (2013) and showed that the transparency of email encryption has no significant impact on the level of trust that the users can have in the system. They have conducted a first study with computer science graduate students having some encryption knowledge, to analyse usability of three email tools (Pwn, Mailvelope and Enigmail plugin for Thunderbird). The results of this study have been used to design their own email tools: an integrated encryption email tool (a modified version of Mailevelope supporting Gmail and using Google Chrome) with two versions (transparent and opaque) and a standalone encryption email tool based on the Message Protector designed by Ruoti et al. These tools have then been tested by cryptography novice webmail students (mainly engineering, science and mathematics students, two business students, one political science student) and three non-student users. The results have revealed that users prefer tools that can be integrated with their webmail client, however they trust more standalone tools than browser extensions. The users group was more student focussed than the general public.

Lerner et al. (2017) developed a prototype called “Confidante,” that interfaces with gmail and is based on Keybase, which is a service that automatically links public keys to corresponding social media accounts. The authors then conducted a case study with lawyers and journalists, who are naturally surrounded with sensitive data. They measured their participants using “*Confidante*” as well as established e-mail encryption software to evaluate the usability of their prototype. The study showed that “*Confidante*” succeeds in improving the usability of PGP by automating key management using social media identities. However, the results also showed that all automation need to be carefully balanced with the remaining security guarantees, which is particularly crucial for users that are concerned with highly sensitive data.

Monson et al. (2018) focussed on a specific usability feature of secure emails that concern secure deletion of emails. The user study revealed the preference of the users to have an email tool that allows shortening the lifetime of their emails rather than just encrypting them.

Ghiglieri et al. (2018) executed a cognitive walkthrough of writing and receiving encrypted email *via* PGP and S/MIME in Thunderbird, in an already prepared setup (i.e., installed software, generated personal key pair, import of personal key). Focusing on cognitive questions, such as “*does the user recognize the possibility to secure mail?*,” “*does the user get feedback for his actions ?*,” “*is the feedback intelligible?*,” they concluded that UI design needs improvements for more visual clarity of security status indication, particularly for encryption and signature of outgoing mail. The main limitation of this work is that it focuses on one MUA only (Thunderbird), only two user testings have been conducted and one user already knows Thunderbird MUA through years of usage. In addition, their assumption that “*software installation, key generation, certificate request, configuration are typical responsibilities for system administration, and as such their pre-configured test environment is a realistic scenario*” might be correct for enterprise use, but is questionable for general users.

Toldsdorf and Iacono (2020) depict challenges for UI design of secure email software beyond key management (e.g., control and recognize security status, security indicators, seamless integration into existing environments) instead of focusing on usability challenges through key management processes. In their work, they present a revised UI scheme for secure email with evident security indication and for seamless integration into the MUA mail environment.

Clark et al. (2018) conducted a study in which they compared currently available technologies for securing e-mail communications. Their comparison considered the dimensions of system architecture design, key management strategy, technical realization, privacy implications and usability. They identified different stakeholder groups (such as industry, academia, personal users, product vendors, law enforcement agencies) of secure e-mail exchange, which all showed to have different expectations and priorities regarding e-mail security. They concluded that usability on a day-to-day scale and unpractical key management procedures remain the major obstacles for wide adoption of e-mail end-to-end encryption in general.

Following this work, in (Clark et al., 2021), Clark et al. conducted a systemization of knowledge that analyses in detail the stakeholders of email communication identified in previous work and their different priorities with respect to security goals, utility, deployability and usability. Moreover, they rated the design of key management approaches and security properties of secure email systems. They concluded that the major obstacle for wide adoption of a particular encryption technology is the diversity of expectations of the stakeholder groups, and that a one-size-fits-all solution appears unrealistic and is unlikely to emerge, due to heterogeneous use cases and different stakeholder needs and interests.

While the approach of Clark et al. is more comprehensive with regard to analysing design patterns of secure mail systems and the diversity of stakeholders, our approach puts the focus on usability issues in the context of specific MUA implementations, rather than the conceptual design of the underlying securitization technology.

To conclude, the main contribution of our usability study compared to previous studies and analysis is:

• We have targeted users from different countries (Germany for Europe and Egypt and Morocco for North Africa) to identify potential cultural differences between Western Europe and North Africa concerning awareness, knowledge and usability difficulties encountered.
• We have targeted young adults having an IT profile, more specifically students and employees working for IT organizations.
• We have chosen to analyse existing MUAs and not Webmails only nor designing new secure email tools.
• We have let the users choose the MUAs they would like to use and test according to their preferences.
• We have also analysed pEp in addition to PGP and S/MIME.
• We have focussed on analysing task complexity of the different MUAs and technologies.
• We have presented a comprehensive description of the tasks and sub-tasks required for using the three security mechanisms (PGP, S/MIME and pEp) in a large set of MUAs. The description of user tasks (and sub-tasks) and the corresponding list of issues found with each MUA features an original contribution to the field.

## Conclusion

This paper provides an original analysis of encryption technology for protecting e-mail communication using Pretty Good Privacy (PGP), Secure Multi-Purpose Internet Mail Extension (S/MIME) and Pretty Easy Privacy (pEp). In this study we have employed various methods to evaluate the usability in terms of task complexity of these technology. To do so, we applied a two-fold approach:

(1) We prepared and launched an online survey to assess the popularity, the degree of deployment and the overall user-perceived usability of these three technologies by a broad audience. Moreover, the online survey allowed us to measure the importance of distinct security goals, such as confidentiality, integrity, authenticity and non-repudiation, for our survey participants. We found that all three technologies struggle with low popularity and very low deployment. However, contrary to the degree of deployment, we found that e-mail security is of high importance to e-mail users. Our results show that 66% of our participants consider e-mail confidentiality as important or very important, thus highlighting the relevance of e-mail encryption. Particularly, we found that e-mail users are even more concerned about identity theft, since 78% of our participants want to make sure that no other person is able to write e-mail on their behalf, and 80% of our participants want to be sure that the content of their e-mail cannot be altered by any other entity while being transferred to the intended recipient. This shows that for many users, signing e-mails and thereby achieving authenticity and integrity of e-mail exchange is more important than achieving confidentiality through encryption.
(2) We secondly conducted user testing, in which participants were asked to install, configure and use PGP, S/MIME and pEp implementations in presence of an evaluator of our research team. Through observing our participants in real-time utilizing these three technologies, we obtained a detailed view of the usability issues encountered by users while trying to put e-mail end-to-end encryption into practice. We observed that for PGP, above all, users are overwhelmed with the manual management of public keys, for both their own key pair as well as the public keys of their communication partners. Moreover, for both PGP as well as S/MIME, users struggle with the setup of the encryption technology in their e-mail software. This is due to hidden configuration options, complex configuration menus, too many manual configuration steps and usage of complex technical terminology. S/MIME particularly lacks implementations for webmail platforms. While pEp successfully simplifies and automates the key management process and uses less tech-savvy language in its user interfaces, this technology struggles with very low popularity (none of our participants ever used it prior to our study). Furthermore, only very few pEp implementations were available at the time of our study, and cross-platform support for Apple MacOS or Apple iOS was missing.

Our results might be considered quite preliminary due to the small sample of participants during the user testing and the limited distribution of the online survey to a few Western European and North African countries. We want to stress that they allow us to uncover the “tip of the iceberg,” exposing the complexity of tasks for using e-mail encryption mechanisms. The findings demonstrate that encryption mechanisms still have severe usability issues that make user tasks difficult to complete. It is important to notice that our study address the usability of tools and tasks users engaged to write encrypted message. The usability results might explain whether tasks are difficult (or not) to accomplish but they cannot explain per se the adoption of the tools. Further investigation is required to investigate the principles that might influence users to adopt secure email encryption solution and/or persuade other users to adopt such as tools.

Given the small number of participants in the user test, we cannot generalize our observations to predict the occurrence of usability problems with other users. Nonetheless, we emphasize that the problems reported by participants are relevant and they provide evidence for identifying tasks that users perceive as difficult.

Our analysis of the mail user agents (MUAs) under test revealed that the implementation of the user interface for the three encryption mechanisms (PGP, S/MIME and pEp) is not standardized. Hence, usability problems had to be analyzed in the context of each MUA. In this work, we have presented a comprehensive description of the tasks and sub-tasks required for actively deploying and using PGP, S/MIME and pEp in a large set of MUAs. Such a description of user tasks and the corresponding list of problems found with each MUAs features an original contribution to the field.

Future work of this research should address a larger panel of participants world-wide in order to determine if other aspects such as age, job occupation, gender, education, culture, geo-politics and others might have an impact on the adoption of encryption mechanisms. We suggest that additional user testing using more sophisticated logging and eye-tracking tools would help to collect detailed information about user performance and cognitive workload.

## Data Availability

The raw data supporting the conclusions of this article will be made available by the authors, without undue reservation.
